# Histological and molecular biology diagnosis of neurocysticercosis in a patient without history of travel to endemic areas – Case report

**DOI:** 10.1051/parasite/2012194441

**Published:** 2012-11-15

**Authors:** C. L’Ollivier, L.M. González, T. Gárate, L. Martin, B. Martha, M. Duong, M. Huerre, B. Cuisenier, L.J.S. Harrison, F. Dalle, A. Bonnin

**Affiliations:** 1 Parasitology department, University Hospital Dijon France; 2 Pathology department, University Hospital Dijon France; 3 Infectious diseases department, University Hospital Dijon France; 4 Parasitology department, Centro Nacional de Microbiología Madrid Spain; 5 Histotechnology and Pathology department, Institut Pasteur Paris France; 6 University of Edinburgh, Royal (Dick) School of Veterinary Medicine, Easter Bush Veterinary Centre, The Sir Alexander Robertson Building, Easter Bush Roslin, Midlothian Scotland

**Keywords:** neurocysticercosis, *Taenia solium*, histology, molecular biology, French patient, neurocysticercose, *Taenia solium*, histologie, biologie moléculaire, patient français

## Abstract

Background: in endemic areas, neurocysticercosis appears mainly as a single, large, spherical and non-enhancing intracranial cyst. Case presentation: an atypical case of neurocysticercosis (NCC) in a French Caucasian, without history of travel to endemic areas, was confirmed by histology and molecular speciation. Imaging was atypical, showing several hook-bearing scolices visible in the cyst, while the serology employed was non-contributary. Conclusions: NCC should be considered when multiple taeniid scolices are observed within the same cystic lesion.

## Abstract (fr)

Contexte : en zone endémique, les lésions kystiques intra-crâniales de neurocysticercose sont classiquement uniques, étendues, sphériques et sans prise de contraste. Cas clinique : un cas atypique de neurocysticercose (NCC) chez un caucasien français n’ayant jamais voyagé en zone d’endémie a été confirmé par histologie et biologie moléculaire. L’imagerie était atypique, montrant plusieurs scolex surmontés de crochets et localisés au sein d’une même lésion kystique. La sérologie était non contributive pour le diagnostic. Conclusions : le diagnostic de NCC doit être évoqué lorsque plusieurs scolex sont observés au sein d’une même lésion kystique.

Neurocysticercosis (NCC) is a pleomorphic taeniid parasitic infection, acquired via carriers of the adult intestinal tapeworm, *Taenia solium*. The infection predominates in less developed countries where NCC is the most frequent cause of parasitic infections of the human central nervous system. Clinical symptoms vary according to localization, viability, number and size of the cyst(s) and the extent of the inflammatory reaction. Seizures, increased intracranial pressure and focal neurological deficits are the most common clinical manifestations.

## Case Presentation

A 69-year-old Caucasian man, from the Burgundy region of France, presented with symptoms of asthenia and headache of a two-year duration. There was neither fever nor seizure and no history of travel. The full blood cell count was unremarkable. Computerized tomography (CT) scans indicated a diffuse right frontal lobe lesion. Magnetic resonance imaging (MRI) with gadolinium injection demonstrated a right frontal tumor on the cerebrifalx, the lateral right ventricular floor and the corpus callosum, with an extending process to the right but not to the left lobe. The lesion measured c. 40 × 30 mm, surrounded by a ring of gadolinium enhancement and a peripheral edema. The MRI revealed some internal necrotic or cystic septations including fluid. No other cystic lesion and no sign of recent hemorrhage were found in the brain.

**Fig. 1.**
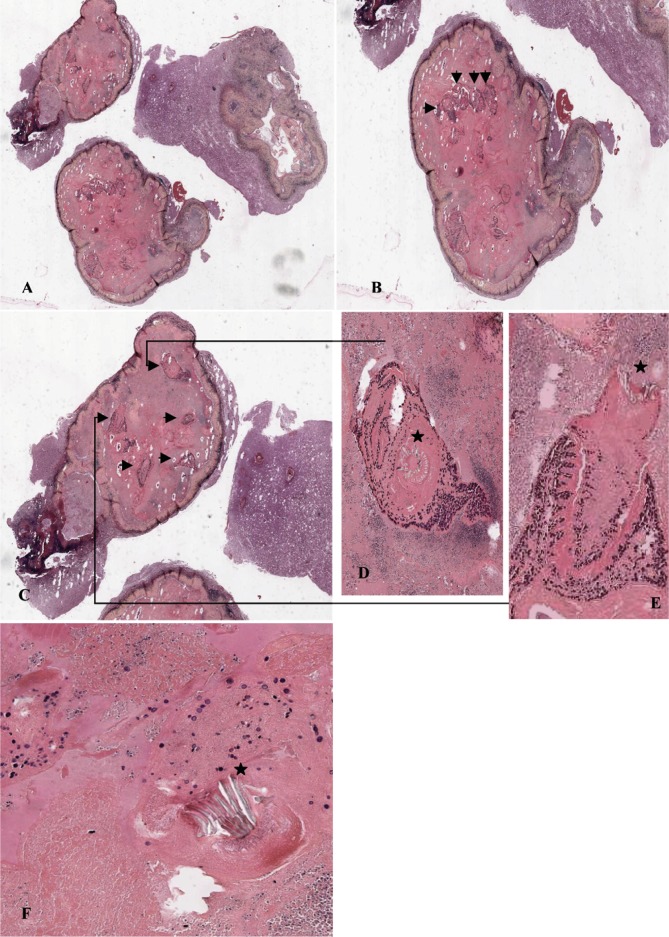
Histological observation of a PAS stained section of the cyst. (A) magnification × 100; (B, C) magnification × 200; (D, E, F) magnification × 600. A: large view of the cerebriform cystic lesion; B, C: higher magnification of two of the various lobes of the cyst including multiples scolices (black arrows); D, E: higher magnification from panel C highlighting tapeworm larvae with hooks (black stars); F: the size of the hooks (double black stars) harboured by the protoscolices was homogeneous, ranging from 100 to 120 μm, that were consistent with *Taenia solium* cysticerci, *i.e.* the larval stage of *T. solium*.

The first hypothesis was a primitive glial tumor and the lesion was resected. Macroscopically, the cystic lesion appeared like a bunch of grapes, the tissue fragments having a yellow appearance, surrounded with a discontinuous band of cerebral tissue. However immuno-histochemical analyses with tumor related markers (GFAP (Glial Fibrillary Acid Protein), S100 protein, EMA, vimentine and synaptophysine) were negative. Finally histological observations excluded a cerebral tumor but highlighted a cystic cavity lined with a fibrotic and inflammatory wall scattered with calcifications and macrophages (Fig. 1). Interestingly, several hook-bearing scolices were visible in the cyst (Fig. 1B, C, D) containing hooks ranging from 100 to 120 μm (Fig. 1E, F). These patterns were consistent with the morphology of *Cysticercus cellulosae*, the most common cysticercus observed in humans. But other cysticercus such as *Taenia saginata*, *Taenia crassiceps*, *Echinococcus multilocularis*, *Cenurosis* were discussed.

Surprisingly, commercially available Enzyme-Linked Immunosorbent Assay (ELISA) designed to detect antiparasite antibody to *Echinococcus granulosus* in serum (Bordier, France) was weakly positive whereas a similar assay for *T. solium* (Biotrin, France) was negative. However, based on clinical, radiographical and histological features, the diagnosis of NCC was retained and the patient received both surgical resection of the cyst and albendazole therapy (15 mg/kg/day, 15 days per month for a nine months period), which was well tolerated.

At the final follow-up consultation, the patient was asymptomatic with no recurrence of symptoms. Repeated MRI revealed the persistence of right frontal lesions up to one year after the diagnosis, reflecting sequellar and non-evolutive lesions. *E. granulosus* ELISA became negative seven months after the lesion was resected.

Molecular identification was retrospectively carried out using previously described PCR protocols (Gonzalez *et al.*, 2000; Gonzalez *et al.*, 2002). These protocols confirmed the species-specific identification of *T. solium* and not *E. granulosus*, as might have been inferred from the serology.

## Conclusions

Accurate diagnosis of NCC is based on the combination of clinical, epidemiological, radiographic, histological and serologic criteria (Del Brutto *et al.*, 2001). Absolute criteria (*i.e.* parasite microscopic observation) allow unequivocal diagnosis of NCC. Major criteria (*i.e.* serological tests) strongly suggest the diagnosis but cannot be used alone to confirm the disease, further their value is closely related to their sensitivity/specificity, which, if insufficient may, as in this case, suggest a misdiagnosis of the species involved. This is where the more specific PCR based diagnostic procedures employed here present an advantage (Gonzalez *et al.*, 2000; Gonzalez *et al.*, 2002). Minor criteria (*i.e.* clinical manifestations) are considered to be nonspecific. Finally, epidemiological criteria refer to circumstantial evidence that favors a cysticercosis/neurocysticercosis diagnosis.

We report herein the diagnosis of NCC in a patient without any apparent epidemiological risk. Indeed, cysticercosis was eradicated in France by a control strategy combining improvements in hygiene, pig husbandry and veterinary sanitary measures. Importantly the patient had no history of travel to endemic areas. However, NCC is now increasingly diagnosed in more developed countries, due to travel/immigration of NCC patients and/or tapeworm carriers from endemic zones (Garcia *et al.*, 2003; Hawk *et al.*, 2005), contact with infectious tapeworm carriers and/or eggs cannot be excluded.

Because of its pleomorphic nature, NCC can be misdiagnosed (Garcia *et al.*, 2003; Lino-Junior Rde *et al.*, 2007). In this case, a tumor was initially suspected as the cyst was in a degenerative stage. However, the parasitic nature of the lesion was confirmed based on the presence of several scolices in the cyst. Although the presence of multiple scolices within the same cystic lesion is infrequent in NCC, the size (100-120 μm) and distribution of the hooks was consistent with *T. solium cysticerci*, the larval stage of *T. solium*.

Cerebral affections involving tapeworm larvae in humans include NCC, cystic echinococcosis and coenurosis (Garcia *et al.*, 2002). Cystic echinococcosis appears mainly as a single, large, spherical and nonenhancing intracranial cyst that was not consistent with the degenerative stage of the observed lesion. *E. granulosus* larval hooks are18 to 23 μm long thus much smaller than the hooks observed in this case (Ahmadi & Dalimi, 2006). Coenurosis is occasionally caused by larvae of *Taenia multiceps* and *Taenia serialis*. The coenurus structure consists of a viscous, fluid-filled bladder containing multiple invaginated scolices, ranging in size from a few millimeters to a few centimeters. The protoscolices have hooklets, of three different types, ranging from 40 to 175 μm again not compatible with the size of the hooks observed in this case. The serological tools employed in this study proved non-contributary to the diagnosis of NCC.ELISA assays are performed in our center as screening tools for the detection of *T. solium* and *E. granulosus* antibodies. When positive, *T. solium* ELISA is secondarily confirmed using a *T. solium* Enzyme-linked immunoelectrotransfer blot (EITB) assay, contributing to the diagnosis of NCC as a major criteria (Hawk *et al.*, 2005; Ito *et al.*, 2006; Sloan *et al.*, 1995). EITB assay was not performed herein since negative results were obtained with the *T. solium* ELISA, indicating the potential contribution of a first line *T. solium* EITB in patients with evocative lesions of NCC.

Retrospectively, the positive *E. granulosus* ELISA was unexpected since NCC was confirmed using the more sensitive and specific PCR based molecular tools. This finding was probably due to the lower sensitivity/ specificity of the *E. granulosus* ELISA (Montenegro *et al.*, 1994) resulting in a cross-reaction. This exemplifies the limited value of serology, especially when weak positivity of the test is observed.

The molecular characterization of *T. solium* using PCR and semi-nested PCR tools confirmed morphological and clinical diagnosis. These tools were used as a retrospective diagnosis, confirming the value of PCR (Gonzalez *et al.*, 2000; Gonzalez *et al.*, 2002) for specific identification of *T. solium* infections in patients with atypical cerebral lesion(s).

Guidelines have been proposed for the management of NCC (Garcia *et al.*, 2002). However, no consensus exists regarding medical management when enhancing lesions are observed with MRI. A recent meta-analysis, comparing the efficacy of albendazole and praziquantel in terms of both resolution of brain cysts and seizure control, reported no superiority of either drugs in the first-line treatment of NC. Finally, medical management remains guided by collateral factors, including drug availability and costs (Garcia, 2008).

*In fine*, NCC must be considered in patients with atypical clinical and neuro-imaging presentations, regardless of travel to endemic areas. In this case, anatomopathological examination and molecular tools were crucial for the diagnosis (i) showing atypical form of NCC with the presence of several larvae in a unique cerebral cystic cavity and (ii) unequivocal PCR-based speciation of the parasite involved.

## References

[R1] Ahmadi N. & Dalimi A. Characterization of *Echinococcus granulosus* isolates from human, sheep and camel in Iran. Infect Genet Evol, 2006, 6 (2), 85–90 16503509 10.1016/j.meegid.2005.01.005

[R2] Del Brutto O.H., Rajshekhar V., White A.C. Jr., Tsang V.C., Nash T.E., Takayanagui O.M., Schantz P.M., Evans C.A., Flisser A., Correa D., Botero D., Allan J.C., Sarti E., Gonzalez A.E., Gilman R.H. & Garcia H.H. Proposed diagnostic criteria for neurocysticercosis. Neurology, 2001, 57 (2), 177–183 11480424 10.1212/wnl.57.2.177 PMC2912527

[R3] Garcia H.H. Antiparasitic drugs in neurocysticercosis: albendazole or praziquantel? Expert Rev Anti Infect Ther, 2008, 6 (3), 295–298 18588494 10.1586/14787210.6.3.295 PMC7021508

[R4] Garcia H.H., Evans C.A., Nash T.E., Takayanagui O.M., White A.C. Jr., Botero D., Rajshekhar V., Tsang V.C., Schantz P.M., Allan J.C., Flisser A., Correa D., Sarti E., Friedland J.S., Martinez S.M., Gonzalez A.E., Gilman R.H. & Del Brutto O.H. Current consensus guidelines for treatment of neurocysticercosis. Clin Microbiol Rev, 2002, 15 (4), 747–756 12364377 10.1128/CMR.15.4.747-756.2002 PMC126865

[R5] Garcia H.H., Gonzalez A.E., Evans C.A. & Gilman R.H. *Taenia solium* cysticercosis. Lancet, 2003, 362 (9383), 547–556 12932389 10.1016/S0140-6736(03)14117-7 PMC3103219

[R6] Gonzalez L.M., Montero E., Harrison L.J., Parkhouse R.M. & Garate T. Differential diagnosis of *Taenia saginata* and *Taenia solium* infection by PCR. J Clin Microbiol, 2000, 38 (2), 737–744 10655377 10.1128/jcm.38.2.737-744.2000 PMC86191

[R7] Gonzalez L.M., Montero E., Puente S., Lopez-Velez R., Hernandez M., Sciutto E., Harrison L.J., Parkhouse R.M. & Garate T. PCR tools for the differential diagnosis of *Taenia saginata* and *Taenia solium* taeniasis/cysticercosis from different geographical locations. Diagn Microbiol Infect Dis, 2002, 42 (4), 243–249 12007441 10.1016/s0732-8893(01)00356-x

[R8] Hawk M.W., Shahlaie K., Kim K.D. & Theis J.H. Neurocysticercosis: a review. Surg Neurol, 2005, 63 (2), 123–132; discussion 132 15680651 10.1016/j.surneu.2004.02.033

[R9] Ito A., Takayanagui O.M., Sako Y., Sato M.O., Odashima N.S., Yamasaki H., Nakaya K. & Nakao M. Neurocysticercosis: clinical manifestation, neuroimaging, serology and molecular confirmation of histopathologic specimens. Southeast Asian J Trop Med Public Health, 2006, 37(Suppl. 3), 74–81 17547057

[R10] Lino-Junior Rde S. Faleiros A.C., Vinaud M.C., Oliveira F.A., Guimaraes J.V., Reis M.A. & Teixeira V.De.P. Anatomopathological aspects of neurocysticercosis in autopsied patients. Arq Neuropsiquiatr, 2007, 65 (1), 87–91 17420834 10.1590/s0004-282x2007000100019

[R11] Montenegro T., Gilman R.H., Castillo R., Tsang V., Brandt J., Guevara A., Sanabria H. Verastegui M., Sterling C. & Miranda E. The diagnostic importance of species specific and cross-reactive components of *Taenia solium*, *Echinococcus granulosus*, and *Hymenolepis nana*. Rev Inst Med Trop Sao Paulo, 1994, 36 (4), 327–334 7732263 10.1590/s0036-46651994000400005

[R12] Sloan L., Schneider S. & Rosenblatt J. Evaluation of enzymelinked immunoassay for serological diagnosis of cysticercosis. J Clin Microbiol, 1995, 33 (12), 3124–3128 8586686 10.1128/jcm.33.12.3124-3128.1995 PMC228657

